# Supplementary material for: The Systems Biology Research Tool: evolvable open-source software
Source: BMC Syst Biol. 2008 Jun 29;2:55. doi: 10.1186/1752-0509-2-55 (PMC2446383; doi:10.1186/1752-0509-2-55)
Supplement: Additional file 1 — SBRT Archive. An archive of the current version of the Systems Biology Research Tool. [file 1752-0509-2-55-S1.zip › sbrt-1.4.0/doc/developers_guide/api/sbrt/shell/io/class-use/InputFile.html]

Uses of Interface sbrt.shell.io.InputFile


|  |  |  |
| --- | --- | --- |
| **Overview** \| **Package** \| **Class** \| **Use** \| **Tree** \| **Deprecated** \| **Index** \| **Help** | |  |
| PREV   NEXT | **FRAMES**    **NO FRAMES**     **All Classes** |  |


---


## **Uses of Interface sbrt.shell.io.InputFile**

| Packages that use InputFile | |
| --- | --- |
| **sbrt.shell.io** | Provides classes and interfaces for reading and writing files. |
| **sbrt.shell.io.fba** | Provides classes and interfaces for reading and writing files relevant to Flux Balance Analysis. |
| **sbrt.shell.mng.fba** | Provides classes and interfaces for creating Flux Balance Analysis process managers. |

Uses of InputFile in sbrt.shell.io

| Subinterfaces of InputFile in sbrt.shell.io | |
| --- | --- |
| `interface` | `MapInputFile<K,V>`             This interface is used to represent input files that contain maps. |
| `interface` | `MapTextInputFile<K,V>`             This interface is used to represent text-based input files that contain maps. |

| Classes in sbrt.shell.io that implement InputFile | |
| --- | --- |
| `class` | `AbstractMapInputFile<K,V>`             This abstract class is a skeleton implemenation of text-based map input files. |
| `class` | `ComiFile<K,V>`             This class is used to read column-oriented map input files. |
| `class` | `ProcessInputFile`             This class is used to represent process input files. |
| `class` | `RomiFile<K,V>`             This class is used to read row-oriented map input files. |
| `class` | `SerializedInputFile<E>`             This class is used to read input files containing serialized objects. |
| `class` | `SerializedMapInputFile<K,V>`             This class is used to read input files containing serialized maps. |
| `class` | `TextInputFile<E>`             This class is used to read input files containing text. |

Uses of InputFile in sbrt.shell.io.fba

| Classes in sbrt.shell.io.fba that implement InputFile | |
| --- | --- |
| `class` | `FluxCapInputFile`             This class is used to read files containing flux caps. |
| `class` | `FluxIntervalInputFile`             This class is used to read flux interval input files. |
| `class` | `RxnNamExprSetInputFile<E extends MathExpr<S>,S>`             This class is used to read files containing a set of reaction name expressions. |

Uses of InputFile in sbrt.shell.mng.fba

| Methods in sbrt.shell.mng.fba that return InputFile | |
| --- | --- |
| `static InputFile<java.util.Set<java.lang.String>>` | `CatalystDeletionManager.getDeletionFile(java.lang.String fileName, CatalyzedFluxome fluxome)`             Returns the catalyst input file with the provided name. |
| `static InputFile<java.util.Set<java.lang.String>>` | `CatalystDeletionManager.getDeletiontFile(ManagerInput<java.lang.String,java.lang.String> input, java.io.PrintWriter out, CatalyzedFluxome fluxome)`             Parses and returns the value for `CatalystDeletionManager.CATALYST_DELETION_FILE` in the provided manager input. |
| `static InputFile<java.util.Map<java.lang.String,java.lang.Double>>` | `SvCdHarFluxVectorManager.getInitialFluxVectorFile(ManagerInput<java.lang.String,java.lang.String> input, java.io.PrintWriter out, Fluxome fluxome)`             Returns the input file containing a collection of initial flux vectors. |
| `static InputFile<java.util.Set<java.lang.String>>` | `RxnDeletionManager.getInputFile(ManagerInput<java.lang.String,java.lang.String> input, java.io.PrintWriter out, Fluxome fluxome)`             Parses and returns the value for `RxnDeletionManager.RXN_DELETION_FILE` in the provided manager input. |
| `static InputFile<java.util.Set<java.lang.String>>` | `RxnDeletionManager.getInputFile(java.lang.String fileName, Fluxome fluxome)`             Returns the reaction name input file with the provided name. |

---


|  |  |  |
| --- | --- | --- |
| **Overview** \| **Package** \| **Class** \| **Use** \| **Tree** \| **Deprecated** \| **Index** \| **Help** | |  |
| PREV   NEXT | **FRAMES**    **NO FRAMES**     **All Classes** |  |


---
